# Chemical composition and antimicrobial activities of the essential oils from three ecotypes of *Zataria multiflora*

**DOI:** 10.4103/0973-1296.75902

**Published:** 2011

**Authors:** K. Zomorodian, M. J. Saharkhiz, M. J. Rahimi, A. Bandegi, G. Shekarkhar, A. Bandegani, K. Pakshir, A. Bazargani

**Affiliations:** 1 *Center of Basic Researches in Infectious Diseases, Shiraz, Iran*; 2 *Departments of Medical Mycology and Parasitology, Shiraz, Iran*; 3 *Department of Horticultural Sciences, Faculty of Agriculture, Shiraz University, Shiraz, Iran*; 4 *Medical Bacteriology and Virology, School of Medicine, Shiraz University of Medical Sciences. Post code 71348-45794, Shiraz, Iran*

**Keywords:** Antimicrobial activity, chemotype, carvacrol, essential oil, linalool, thymol, *Zataria multiflora*

## Abstract

**Background:**

*Zataria multiflora* Boiss. is a traditional and popular spice in Iran. The effects of 3 ecotypes (ECTPs) of *Z. multiflora* essential oils (EOs) against most common causes of food-borne and nosocomial infections were evaluated.

**Materials and Methods:**

The antimicrobial activities of the EOs were examined by broth microdilution method as recommended by the Clinical and Laboratory Standards Institute (CLSI). The chemical compositions of the EOs from 3 ECTPs of *Z. multiflora* have been analyzed by gas chromatography–mass spectrometry.

**Results:**

Analysis of the EOs indicated that 3 chemotypes were present in *Z. multiflora*, including carvacrol, thymol-carvacrol, and linalool, whereas previous studies have only found carvacrol and thymol. Inhibition studies showed that the tested EOs entirely inhibited the growth of yeasts at concentrations of less than 1 *μ*L/mL. Moreover, the oils exhibited significant bacteriostatic and bactericidal activities against Gram-positive and Gram-negative bacteria at concentrations ranging from 0.12 to 8 *μ*L/mL.

**Conclusion:**

These results suggest that the EOs from *Z. multiflora* should be investigated further for possible use in antimicrobial products and food preservatives.

## INTRODUCTION

*Zataria multiflora* Boiss. with the common Persian name “Avishan-e Shirazi” is a thyme-like essential oil (EO)-bearing plant that belongs to the Lamiaceae family and grows extensively wild in the central and southern parts of Iran, Pakistan, and Afghanistan.[1] The dry aerial parts of the plant have been used for their flavor and preservative properties in the food products industry.[2] In Iran, *Z. multiflora* is mainly used in traditional folk remedies for its antiseptic, analgesic, and carminative (antiflatulence and intestine-soothing) properties.[1] It also has been reported that the EOs and extracts of *Z. multiflora* can stimulate innate immunity[3] and have antibacterial and antifungal activities.[1,4,5] In addition, *Z. multiflora* EOs have been shown to cause inhibitory effects against radial fungal growth and aflatoxin production by *Aspergillus flavus* in cheese.[2] Moreover, the oil and extracts of *Z. multiflora* successfully inhibited the growth of bacteria associated with gastrointestinal infections, including *Staphylococcus aureus*,[6] enterohemorrhagic *Escherichia coli*,[7] *Salmonella* Typhi and Paratyphi,[8] and *Shigella flexneri* and *Bacillus cereus*.[8,9] In the past 2 decades, the emergence of resistance to various antibiotics has accelerated dramatically. Methicillin-resistant *S. aureus* (MRSA), vancomycin-resistant *Enterococcus* (VRE) species, third-generation cephalosporin-resistant (TGCsR) *Escherichia coli*, imipenem and quinolone-resistant *Pseudomonas aeruginosa*, antibacterial-resistant *Salmonella* and *Shigella* species, as well as azole-resistant *Candida* species are the top resistant pathogens responsible for food-borne or nosocomial infections.[10,11] To overcome antibiotic resistance, there is a great tendency toward using natural products and phytochemicals in the medicine and food industries. EOs, especially with known antibacterial effects, have the potential to be used in the food industry as a preservative, for spoilage prevention, and to increase the shelf life of products. Therefore, determining the antimicrobial properties of EOs might help to overcome microorganism resistance to antibiotics.

To the best of our knowledge, only a few published reports are available regarding the antimicrobial effects of the *Z. multiflora* EOs, especially against the above-mentioned resistant microorganisms. In the present study, the chemical constituents of 3 ecotypes (ECTPs) of *Z. multiflora* were studied and their components were compared with each other and to previously reported data. In addition, the antimicrobial effects of these ECTPs were evaluated against standard strains and clinical isolates of nosocomial infections as well as some food-borne agents.

## MATERIALS AND METHODS

### Collection of plant material

Three ECTPs of *Z. multiflora* were used to extract the EOs. The plant ECTPs from which the EOs were extracted were collected from 3 different ecologic areas. The aerial parts of ECTP *A*, *B*, and *C*, including flowers were obtained from wild plants in Lamerd, Darab, and Zarghan regions in Fars province, Iran, respectively. Lamerd and Darab are about 855 m above the mean sea level with warm-dry climate, whereas Zarghan is about 1602 m and has a semi-arid climate.

The plant species were identified and authenticated by Dr. A.R. Khosravi, a plant taxonomist, at Shiraz University, Herbarium, Shiraz, Iran. Voucher specimens (No. 24984, 24985, and 24986) were deposited in the herbarium.

### EO preparation

At full flowering stage, the aerial parts of the ECTPs were hydrodistillated for 2.5 h, using an all-glass Clevenger-type apparatus, according to the method outlined by the British Pharmacopoeia.[12] The sample oils were dried over anhydrous sodium sulfate and stored in sealed vials at 4°C before gas chromatography and gas chromatography–mass spectrometry (GC–MS) analysis.

### EO analysis by gas chromatography–mass spectrometry

The EOs were analyzed by GC–MS. The analysis was carried out on a Thermoquest–Finnigan Trace GC–MS instrument equipped with a DB-5 fused silica column (60 m ×0.25 mm i.d., film thickness 0.25 mm). The oven temperature was programmed to increase from 60°C to 250°C at a rate of 4°C/min and finally held for 10 min; transfer line temperature was 250°C. Helium was used as the carrier gas at a flow rate of 1.1 mL/min with a split ratio equal to 1/50. The quadrupole mass spectrometer was scanned over the 35–465 amu with an ionizing voltage of 70 eV and an ionization current of 150 mA.

GC- flame ionization detector (FID) analysis of the oil was conducted using a Thermoquest-Finnigan instrument equipped with a DB-5 fused silica column (60 m × 0.25 mm i.d., film thickness 0.25 mm). Nitrogen was used as the carrier gas at the constant flow of 1.1 mL/min; the split ratio was the same as for GC–MS. The oven temperature was raised from 60°C to 250°C at a rate of 4°C/min and held for 10 min. The injector and detector (FID) temperatures were kept at 250°C and 280°C, respectively. Semi-quantitative data were obtained from FID area percentages without the use of correction factors.

### Identification of EO components

Retention indices (RI) were calculated by using retention times of *n*-alkanes (C6–C24) that were injected after the oil at the same temperature and conditions. The compounds were identified by comparing their RI with those reported in the literature, and their mass spectrum was compared with those reported in Wiley Library.[13]

### Determination of antimicrobial activities

#### Microorganisms

The antifungal activities of the EO against 5 American Type Culture Collection (ATCC) strains of fungi, including *Candida albicans* (ATCC 10261), *C. tropicalis* (ATCC 750), *C. krusei* (ATCC 6258), *C. glabrata* (ATCC 90030), and *C. parapsilosis* (ATCC 4344) were determined. In addition, the antimicrobial activities of the EO against 40 clinical isolates of yeasts identified by polymerase chain reaction–restriction fragment length polymorphism (PCR–RFLP) were also examined.[14,15] The antibacterial activities of the EO against standard species of *S. aureus* (ATCC 25923 and ATCC 700698), *E. faecalis* (ATCC11700), *Escherichia coli* (ATCC 25922), enterohemorrhagic *E. coli* (ATCC 43894), *P. aeruginosa* (ATCC 27853), *Sh. flexneri* (NCTC 8516), *Salmonella enterica* subsp. *enterica* (ATCC 14028), and clinical isolates of *S. aureus, E. faecalis, *E. faecium*, E. coli,* and *P. aeruginosa* collected from the Dr. Faghihi Hospital (Shiraz, Iran) were also determined in this study. The susceptibility of all clinical isolates of bacteria and fungi against select antibiotics were examined by microdilution and disk diffusion methods.[16,17]

#### Determination of minimum inhibitory concentration

MICs were determined using the broth microdilution method recommended by the CLSI with some modifications.[16,17] Briefly, for determination of antimicrobial activities against yeast, serial dilutions of the EOs (0.007–32.0 *μ*L/mL) were prepared in 96-well microtiter plates using RPMI-1640 media (Sigma, St. Louis, MO, USA) buffered with MOPS (Sigma). To determine the antibacterial activities, serial dilutions of the EOs (0.03–128.0 *μ*L/mL) were prepared in Muller–Hinton media (Merck, Darmstadt, Germany). Test yeasts or bacteria strains were suspended in media and the cell densities were adjusted to 0.5 McFarland standards at 530 nm wavelength using a spectrophotometeric method (this yields stock suspension of 1–5 × 10^6^ cells/mL for yeasts and 1–1.5 × 10^8^ cells/mL for bacteria). Working inoculums (0.1 mL) was added to the microtiter plates, which were incubated in a humid atmosphere at 30°C for 24–48 h (yeast) or at 37°C for 24 h (bacteria). Uninoculated medium (200 μL) was included as a sterility control (blank). In addition, growth controls (medium with inoculums but without EO) were also included. The growth in each well was compared with that of the growth in the control well. MICs were visually determined and defined as the lowest concentration of the EO produced ≥50% growth inhibition for fungi and ≥95% growth reduction for bacteria compared with the growth in the control well. Each experiment was performed in triplicate.

In addition, media from wells with fungi showing no visible growth were further cultured on Sabouraud dextrose agar (Merck, Darmstadt, Germany) and from wells with bacteria showing no visible growth on Muller-Hinton agar (Merck, Darmstadt, Germany) to determine the minimum fungicidal concentration (MFC) and minimum bactericidal concentration (MBC). MBCs and MFCs were determined as the lowest concentration yielding no more than 4 colonies, which corresponds to a mortality of 98% of the microbes in the initial inoculums.

## RESULTS

The qualitative and quantitative compositions of the EOs of the full flowering, aerial parts of *Z. multiflora* ECTPs are presented in Table 1. GC–MS analyses showed that the main constituents of the EOs from ECTPs *A* and *B* were carvacrol (82.7%) and thymol-carvacrol (38.88%/27.16%), whereas that of ECTP *C* was linalool (87.35%).

**Table 1:** Chemical components (%) of the essential oils distilled from 3 ecotypes of Zataria multiflora

Components	RI^a^	Chemotypes peak area		
		ECTP A	ECTP B	ECTP C
alpha-Thujene	929	0.74	0.72	0.11
alpha-Pinene	939	2.65	2.11	5.10
Camphene	954	0.14	0.03	0.00
1-Octene-3-one	972	0.00	0.00	0.44
Sabinene	976	0.00	0.00	0.17
beta-Pinene	982	0.35	0.25	0.57
Myrcene	989	1.63	1.38	0.00
trans-Dehydroxy-linalool oxide	992	0.00	0.00	0.18
cis-DEHYDROXY-linalool oxide	1007	0.00	0.00	0.37
alpha-Phellandrene	1007	0.90	0.08	0.00
delta-3-Carene	1014	0.13	0.11	0.00
alpha-Terpiene	1019	1.21	0.5	0.00
p-Cymene	1028	14.89	4.7	0.00
Limonene	1031	15.76	0.12	0.00
1,8-Cineol	1035	0.21	0.19	0.00
Z-beta-Ocimeme	1044	0.54	0.21	0.00
gamma-Terpienne	1060	5.80	2.19	0.00
cis-Linalool oxide	1076	0.00	0.00	1.56
trans-Sabinene hydrate	1079	0.00	0.05	0.00
alpha-Terpinolene	1090	0.00	0.59	0.00
trans-Linalool oxide	1091	0.00	0.00	0.92
Linalool	1098	0.70	0.48	87.35
Octyl acetate	1178	0.00	0.00	0.38
Terpin-4-ol	1184	0.42	0.00	0.00
Dihydrocarvone	1216	0.27	0.00	0.00
E-isocitral	1220	0.00	0.00	0.97
Carvacrol methyl ether	1243	1.35	1.47	0.00
Linalyl acetate	1253	0.00	0.00	0.18
Thymol	1292	38.88	0.10	0.00
Carvacrol	1319	27.16	82.7	0.00
Carvacrol acetate	1373	0.98	0.91	0.00
beta-Caryophyllene	1435	1.22	1.31	1.11
alpha-Humulene	1453	0.14	0.34	0.00
allo-Aromadendrene	1467	0.00	0.25	0.00
Bicyclogermacrene	1506	0.00	0.14	0.25
Caryophyllene oxide	1598	6.31	5.36	0.00

ECTP: ecotype., Major components of the essential oils are indicated by bold letters, aRetention Indices on DB-5 column

The antibacterial activities of *Z. multiflora* EOs against the tested bacteria are shown in Table 2. The EOs inhibited the growth of all Gram-positive cocci at concentrations of 0.12–4 μL/mL. Furthermore, the EOs exhibited bactericidal activity (MBC) for all of the above-mentioned Gram-positive cocci at concentrations ranging from 1 to 8 μL/mL. Among the studied EOs, ECTP *A* had the highest inhibitory effect against the Gram-positive cocci with minimum inhibitory concentrations (MICs) about half of those of ECTPs *B* and *C*. No significant differences in inhibitory concentrations were found between antibiotic-resistant and -susceptible strains. All of the *E. coli* strains were susceptible to *Z. multiflora* EOs at concentrations of 0.12-8 μL/mL, while the EOs only inhibited the growth of about half of the isolates of *P. aeruginosa* at concentrations of 2-128 μL/mL. In addition, the EOs had bactericidal activity against all of the strains of *E. coli* and some strains of *P. aeruginosa* at concentrations of 0.12-8 μL/mL and 4-128 μL/mL, respectively.

**Table 2:** Antibacterial activity (MIC and MBC) of essential oils distilled from *Zataria multiflora’s* ecotypes

Bacteria (number of strains)		ECTP A		ECTP B		ECTP C	
		MIC90 (uL/ mL) GM^a^ (range)	MBC (uL/mL) GM^a^ (range)	MIC90 (uL/ mL) GM^a^ (range)	MBC (uL/mL) GM^a^ (range)	MIC90 (uL/ mL) GM^a^ (range)	MBC (uL/mL) GM^a^ (range)
Gram positive	Methicillin-resistant *S. aureus* (6)	0.557 (0.12–1)	2.83 (1–4)	1.414 (1–2)	5.04 (2–8)	1.414 (1–2)	3.56 (2–8)
	Methicillin-sensitive *S. aureus* (6)	0.442 (0.12–1)	2.244 (1–4)	0.89 (0.5–1)	3.174 (2–4)	1.122 (1–2)	4.49 (2–8)
	Vancomycin-resistant *E. faecalis* (2)	0.5 (0.5)	1 (1)	2.828 (2–4)	2.828 (2–4)	1.414 (1–2)	2 (2)
	Vancomycin-sensitive *E. faecalis* (5)	0.435 (0.25–1)	1.148 (1–2)	1.319 (1–2)	1.64 (2–4)	2 (1–4)	4 (4)
	Vancomycin-resistant *E. faecium* (4)	0.42 (0.25–0.5)	1 (1)	0.84 (0.5–2)	2.378 (1–4)	1.19 (1–2)	6.72 (4–8)
Gram negative	Third-generation cephalosporin-resistant *E. coli* (6)	1.148 (0.5–2)	1.319 (0.5–4)	0.757 (0.5–2)	1 (0.5–4)	3.031 (2–8)	4.59 (2–8)
	Third-generation cephalosporin-sensitive *E. coli* (5)	0.659 (0.12–1)	0.659 (0.12–1)	1.515 (0.25–2)	2.297 (0.25–4)	2.297 (1–4)	3.031 (2–4)
	Multidrug-resistant *P. aeruginosa* (6)	64 to >128	64–>128	32–>28	64–>128	32 to >128	64 to >128
	Sensitive strain *P. aeruginosa* (5) (5)	64 to >128	>128	32–>128	32–>128	32 to >128	64 to >128
	*Sh. flexneri* NCTC 8516	>0.12	>0.12	0.06	0.12	0.5	1
	*S. enterica* ATCC 14028	>0.12	>0.12	0.25	0.25	2	2

ECTP: ecotype, MIC: Minimum inhibitory concentration, MBC: Minimum bactericidal concentration

a Geometric mean

The antimicrobial activities of *Z. multiflora* EOs against yeasts are shown in Table 3. For the clinical and standard yeasts tested, the MICs for the EOs were 0.003–0.5 μL/mL. Among the examined EOs, ECTP *A* had the highest fungicidal activity with MFC values ranging from 0.03 to 0.25 μL/mL, followed by ECTP *B* (MFC: 0.03-1 μL/mL) and ECTP *C* (MFC: 0.25-4 μL/mL).

**Table 3:** Antifungal activity (MIC and MFC) of the essential oils of *Zataria multiflora’s* ecotypes

	ECTP A (μL/mL)	ECTP B (μL/mL)	ECTP C (μL/mL)	Fluconazole (μg/mL)	Itraconazole (μg/mL)
Isolates (number)	MIC50 GM^a^ (Range)	MIC50 GM^a^ (Range)	MIC50 GM^a^ (Range)	R^b^ ≥ 64	R^b^ ≥ 1
	MIC90 GM^a^ (Range)	MIC90 GM^a^ (Range)	MIC90 GM^a^ (Range)	S–DD^c^ : 16–32	S–DD^c^ : 0.25–0.5
	MFC GM^a^ (Range)	MFC GM^a^ (Range)	MFC GM^a^ (Range)	S^d^ ≤ 8	S^d^ ≤ 0.12
*C. albicans* (n=26)	0.012 (0.0075–0.03)	0.021 (0.0075–0.06)	0.106 (0.03–0.5)	7	11
	0.023 (0.0075–0.12)	0.049 (0.015–0.25)	0.29 (0.06)–1)	1	2
	0.052 (0.015–0.25)	0.118 (0.03–0.5)	0.765 (0.25–4)	18	13
*C. tropicalis* (n=3)	0.023 (0.015–0.06)	0.037 (0.03–0.06)	0.155 (0.06–0.25)	2	2
	0.047 (0.03–0.12)	0.075 (0.06–0.12)	0.391 (0.12–1)	1	1
	0.076 (0.03–0.25)	0.153 (0.06–1)	1 (0.5–2)	0	0
*C. dubliniensis* (n=4)	0.01 (0.0075–0.03)	0.012 (0.0075–0.015)	0.25 (0.015–0.25)	1	1
	0.015 (0.0075–0.03)	0.03 (0.03)	0.2 (0.03–0.5)	0	1
	0.035 (0.03–0.06)	0.06 (0.03–0.12)	0.84 (0.25–2)	3	2
*C. glabrata* (n=5)	0.0075 (0.0075)	0.007 (0.003–0.015)	0.034 (0.015–0.6)	0	0
	0.0098 (0.0075–0.015)	0.015 (0.0075–0.03)	0.068 (0.03–0.12)	0	4
	0.035 (0.03–0.06)	0.12 (0.06–0.25)	0.287 (0.25–0.5)	5	1
*C. parapsolosis* (n=5)	0.0098 (0.0075–0.03)	0.011 (0.0035–0.015)	0.045 (0.03–0.12)	0	0
	0.015 (0.0075–0.03)	0.017 (0.0035–0.3)	0.091 (0.06–0.25)	0	0
	0.022 (0.00750.06)	0.08 (0.03–0.12)	0.57 (0.5–1)	5	5
*C. krusei* (n=1)	0.015	0.03	0.12	0	0
	0.06	0.06	0.5	1	0
	0.06	0.06	1	0	1
Trichosporon (n=1)	0.015	0.015	0.5	1	1
	0.03	0.03	1	0	0
	0.03	0.03	1	0	0

MIC: Minimum inhibitory concentration, MFC: Minimum fungicidal concentration

a Geometric mean

b Resistant

c Susceptible–dose dependent

d Susceptible

## DISCUSSION

The composition of the EOs may vary greatly depending on the geographic region from which they are collected. The chemical composition of *Z. multiflora* EOs has already been reported.[1,3,18,19] Based on GC–MS analyses of the oils collected from different regions of Fars province in this study, 3 distinct chemotypes were identified. Similar to the majority of earlier studies,[9,19] we found carvacrol (82.7%) as the major ingredient of the EO extracted from the aerial parts of *Z. multiflora* collected from the Lamerd region (ECTP *A*). The higher concentration of carvacrol in this study as compared to those of previous reports[9,19] may reflect variations due to geographical location from which the plants were collected. Alternatively, it may be due to differences in collecting fresh plants from fields as was done in this study vs purchasing them from herbal stores as in some studies, which may lead to the loss of parts of their volatile compounds.

Similar to previous reports,[1,3,20] a combination of 2 phenolic compounds, including carvacrol and thymol as the main constituents, was identified in the *Z. multiflora* EO collected from the Darab region (ECTP *B*), whereas very low amounts of thymol (<0.1%) were detected in the EO from the Lamerd region (ECTP *A*). Moreover, we found linalool (87.35%) as the main component of the EO collected from the Zarghan region (ECTP *C*). The identification of a linalool chemotype (ECTP *C*) in this study is in agreement with the study by Mohagheghzadeh *et al*. that reported high concentrations of linalool (60.39%) and linalyl acetate (8.55%) in the *Z. multiflora* EO collected from Kolahghazi (near Isfahan, Iran), which introduced an alcoholic chemotype as well as phenolic ones.[21]

*Candida* species are associated with mucocutaneous infections and currently are considered as the fourth most common causes of bloodstream infections. During the past several years, resistance to traditional triazole antifungal drugs, such as itraconazole and fluconazole, among clinical isolates of *Candida* has increased dramatically, justifying demands for novel antifungals.[22] The MICs and MFCs of the EOs of the 3 ECTPs of *Z. multiflora* were determined for the examined yeasts, showing strong anti-*Candida* activities with MIC values ranging from 0.007 to 0.5 μL/mL. This finding is similar to that of the study by Mahboubi *et al*. who reported strong anti-Candida activity of *Z. multiflora* EO with high thymol and carvacrol concentrations.[20] The lower MICs and MFCs of the examined EOs in this study as compared with that of a previous report[23] may be due to differences in the oil constituents or in the method used to assay antimicrobial activity (they used a macrodilution method and Sabouraud dextrose broth instead of a microdilution method and RPMI). Although the EO concentrations that caused inhibition were higher than those of antifungal drugs, the results are of interest because the EO is a mixture of components and not a pure compound.[23] As the 0.1% *Z. multiflora* cream was successfully used in the treatment of vaginal candidiasis,[24] the tested EOs may be potentially valuable as natural treatments of mucocutaneous candidiasis and geotrichosis.

Based on previous epidemiologic studies, *E. coli* O157:H7 accounts for many food-borne outbreaks in different countries. In this study, the EOs inhibited the growth of this strain at concentrations of 0.12, 0.25, and 2 μL/mL for ECTPs *A, B, and C*, respectively, which can be best compared to the study reported by Fazlara *et al*. on the same strain.[6] Similar to the study by Abbasgholizadeh *et al*., the EOs showed bactericidal effects against the clinical isolates of TGCsR and TGCsS *E. coli* at concentrations ranging from 0.12 to 8 μL/mL.[25] In addition, the EOs exhibited inhibitory and bactericidal activities against *S. entrica* and *Sh. flexneri* at concentrations ranging from >0.12 to 2 μL/mL. The antibacterial activity of ECTP B in this study is most comparable to that of Dakhili *et al*. who reported significant antibacterial effects against S. Typhimurium from the EO of *Z. multiflora*, which is rich in thymol and carvarole.[26] Moreover, they reported that *Z. multiflora* EO had strong antibacterial activity against *S. Typhimurium*, which is most comparable with the ECTP B in the present study.[26]

*Staphylococcus aureus* is one of the 4 most common causes of nosocomial infections, often causing postsurgical wound contamination. It is also considered as one of the main etiologic agents of food-borne infections.[11] In addition, there is major concern about this species due to the fast development of methicillin resistance. Similar to previous reports,[27] the growth of the standard and clinical isolates of MRSAs and MSSAs was inhibited by ECTPs *A, B*, and *C* of *Z. multiflora* EOs at concentrations of 0.55 to 1.41 μL/mL, respectively. In another study, Mahboubi and Ghazian found a chemotype of the *Z. multiflora* oil rich in thymol/carvacrol that significantly inhibited the growth of both MRSA and MSSA at concentrations ranging from 0.06 to 1 μL/mL, which is comparable to the ECTP B results found in this study.[18] Interestingly, the tested EOs had bactericidal activities and killed all of the *S. aureus* at concentrations less than 8 μL/mL. Moreover, it has been shown that the EO significantly prevented production of staphylococcal enterotoxin C during the manufacturing process of white brined cheese at concentrations as low as 5 μL/100 mL[28]; hence, it might be used as a preservative additive in the food industry.

Vancomycin-resistant *E. faecium* is a problematic pathogen with few treatment options. All the tested ECTPs exhibited strong antimicrobial activities against vancomycin-resistant *E. faecium* as well as both vancomycin-resistant E. faecalis (VREf) and vancomycin-sensitive E. faecalis (VSEf). The MICs of ECTP A of *Z. multiflora* EO against VREfs and VSEfs in this study were much lower than those reported by Ravanshad *et al*., which used commercial EO and a macrodilution method.[29]

Among Gram-negative bacteria, *P. aeruginosa* appears to be the least sensitive to the EOs. In the present study, the tested EOs (ECTPs of *Z. multiflora*) killed 30%–50% of the susceptible and multidrug-resistant strains of *P. aeruginosa* at concentrations of up to 128 μL/mL (with MIC values ranging from 32 to 128 μL/mL).

The MICs and MBCs of the EOs against the examined Gram-negative bacteria were almost the same, whereas the MBCs of Gram-positive bacteria were 2–4 times higher than their corresponding MICs. One of the main characteristics of EOs is their hydrophobicity, which enables their incorporation into the cell membrane.[17] Among the tested ECTPs, ECTP *A* had the lowest MICs followed by ECTP *B* and ECTP *C* against both susceptible and resistant strains. ECTPs *A* and *B* were both rich in phenolic monoterpenes, including carvacrol and thymol. It has been shown that these phenolic monoterpenes have hydroxyl groups at different positions around the phenolic ring and exhibit their antimicrobial activities through disruption of the cytoplasmic membrane, which leads to leakage of ions and ATP.[30]

## CONCLUSION

Among the studied ECTPs, ECTP A with high concentration of carvacrol showed better antimicrobial activities than ECTP B and C. As the food industry tends to reduce the use of chemical preservatives in the food products, the EO of *Z. multiflora* with potential active antimicrobial properties might be considered as a natural source for the maintenance or extension of the shelf life of products. In addition, delectable taste of the EO at the concentrations needed for antimicrobial properties was a bonus to its antimicrobial effects. On the other hand, these EOs might also be considered for developing antibiotics and disinfectants for controlling infections caused by nosocomial pathogens. As these tests have all been done *in vitro*, the next step maybe is to further investigate in animal models to see if infection can be inhibited by these EOs.
